# High-Level Acquisition of Maternal Oral Bacteria in Formula-Fed Infant Oral Microbiota

**DOI:** 10.1128/mbio.03452-21

**Published:** 2022-01-18

**Authors:** Shinya Kageyama, Michiko Furuta, Toru Takeshita, Jiale Ma, Mikari Asakawa, Yoshihisa Yamashita

**Affiliations:** a Section of Preventive and Public Health Dentistry, Division of Oral Health, Growth and Development, Faculty of Dental Science, Kyushu University, Fukuoka, Japan; b OBT Research Center, Faculty of Dental Science, Kyushu University, Fukuoka, Japan; Rutgers, The State University of New Jersey

**Keywords:** 16S rRNA, PacBio Sequel II, breastfeeding, mother-to-infant transmission, oral microbiota

## Abstract

The influx of maternal oral microbes is considered to play an important role in the acquisition and development of infant oral microbiota. In this study, we examined tongue swab samples from 448 mother-infant pairs at 4-month checkups. The bacterial composition of each sample was determined using PacBio single-molecule long-read sequencing of the full-length 16S rRNA gene and the amplicon sequence variant (ASV) approach. Although the infant oral microbiota was distinctly different from the mother oral microbiota, ASVs shared with their biological mother accounted for a median relative abundance of 9.7% (range of 0.0 to 99.3%), which was significantly higher than that of ASVs shared with unrelated mothers. This shared abundance was strongly associated with the feeding method of infants rather than their delivery mode or antibiotic exposure, and formula-fed infants had higher shared abundance than exclusively breastfed infants. Our study presents strain-level evidence for mother-to-infant transmission of oral bacteria and suggests that colonization of maternal oral bacteria is higher in formula-fed infants.

## INTRODUCTION

Numerous microbes inhabit the human oral cavity and form a complex microbial ecosystem. A large number of studies have reported an association between oral microbiota and oral diseases, such as dental caries and periodontitis. Moreover, the oral microbiota has recently attracted attention as a microbial source for the digestive tract and respiratory system, and it is of key relevance to diseases of these distant organs (1–5). For instance, several studies have suggested that bacteria which usually inhabit the oral cavity are detected in abundance in the gut microbiota of patients with liver cirrhosis, inflammatory bowel diseases, and colorectal cancer (6–8). Oral bacteria were also detected in the lungs, and our prospective cohort study of nursing home residents demonstrated that the bacterial composition of tongue microbiota, dominated by *Prevotella* and *Veillonella* species, was associated with pneumonia-related death (9–11). Although the causal role of oral microbiota is poorly understood, it is vital to study the oral microbiota to elucidate the etiologies of systemic and oral diseases.

After birth, a newborn’s oral cavity is constantly exposed to extensive and diverse bacteria. The initial formation of the oral indigenous microbiota reportedly begins within the first 6 weeks of life, and Streptococcus species rapidly dominates the oral cavity during this stage (12). Subsequently, the oral microbiota diversifies and develops with age through the acquisition of oral commensal bacteria, which are generally detected in the oral cavity of adults (13, 14). In this process, the maternal oral microbiota appears to play an important role as the major source of the infant oral microbiota. Several previous studies have shown that the microbial profiles of oral microbiota in mother-child pairs are more similar than those of unrelated mother-child pairs (15–17). However, the fidelity of oral microbial transmission from a mother to an infant is poorly understood because of the difficulty in tracking microbes across individuals. Although several factors, including delivery mode (18–20), feeding method (19, 21, 22), antibiotic usage (19), and family smoking status (23) have been reported to affect infant oral microbiota, their effects on microbial transmission have never been studied. The elucidation of the microbial acquisition process can eventually facilitate the creation of a novel approach to inducing healthy development of oral microbiota and preventing oral microbiota-related diseases.

In this study, we examined tongue swab samples collected from 448 mother-infant pairs at 4-month checkups. We determined the bacterial composition of each sample using PacBio single-molecule long-read sequencing of the full-length 16S rRNA gene and the amplicon sequence variant (ASV) approach to precisely evaluate the microbial transmission between each mother and her infant. PacBio long-read sequencing provides highly accurate sequences using the circular consensus sequencing (CCS) mode, which repeatedly determines the template DNA sequences based on long sequencing potential (24). Full-length analysis of the 16S rRNA gene containing nine hypervariable regions and the ASV approach, which resolves differences as little as one nucleotide, can be used to discriminate between bacterial species or strains with high resolution (25). This study aimed to use these approaches to evaluate oral microbial transmission from a mother to an infant in early infancy and to identify the factors influencing transmission.

## RESULTS

### Characteristics of subjects and full-length 16S rRNA gene sequences.

We examined 448 infants (217 males and 231 females) and 444 mothers (including four mothers of twins) who visited for 4-month checkups. Detailed characteristics of the infants are presented in Table 1. At the time of the checkup, 74.6% of the infants were aged ≥4 full months (≥120 days), and 25.4% were aged less than 4 months. Most infants (80.4%) were vaginally delivered, and 19.6% were delivered via cesarean section. More than half (57.2%) of the infants were exclusively breastfed, and 13.5% were exclusively formula-fed. Only 4.5% of the infants used antibiotics within a month prior to the checkup. In total, 892 samples were analyzed by the full-length 16S rRNA gene amplicon analysis using PacBio Sequel II, and a final total of 4,858,255 denoised reads (5493.4 ± 1730.2 reads per infant sample and 5399.1 ± 2281.9 reads per mother sample) and 11,044 ASVs were obtained. The rarefaction curves for the number of ASVs approached a plateau in each sample (Fig. S1 in the supplemental material). Of all ASVs, 10,710 ASVs (99.6% of all reads) exhibited ≥98.5% identity with the reference sequences in the expanded Human Oral Microbiome Database (eHOMD; 26) and were clustered into 279 operational taxonomic units (OTUs).

**TABLE 1:** Characteristics of infants

Characteristic	Subjects (%)
Sex	
Female	231 (51.6)
Male	217 (48.4)
Age	
3 mo	113 (25.4)
4 mo	312 (70.1)
≥5 mo	20 (4.5)
Feeding method	
Breastfeeding	255 (57.2)
Mixed-feeding	131 (29.4)
Formula-feeding	60 (13.5)
Delivery mode	
Vaginal	360 (80.4)
Caesarean-section	88 (19.6)
Antibiotic use	
None	428 (95.5)
Use within one mo	20 (4.5)
Family smoking	
Without	288 (64.3)
With current smoker	160 (35.7)
Gestational age	
≥37 wks	422 (95.3)
<37 wks	21 (4.7)
Birth wt	
≥2,500 g	411 (91.7)
<2,500 g	37 (8.3)
Current wt	
Low	62 (13.9)
Normal	314 (70.6)
High	69 (15.5)
Kaup index	
<16	101 (22.7)
≥16 and <18	241 (54.2)
≥18	103 (23.1)

10.1128/mbio.03452-21.1 FIG S1 Rarefaction curves for a number of observed ASVs per sample. Download FIG S1, TIF file, 0.6 MB. Copyright © 2022 Kageyama et al. 2022 Kageyama et al. https://creativecommons.org/licenses/by/4.0/ This content is distributed under the terms of the Creative Commons Attribution 4.0 International license.

### Microbial link between mother and infant microbiota.

**FIG 1:**
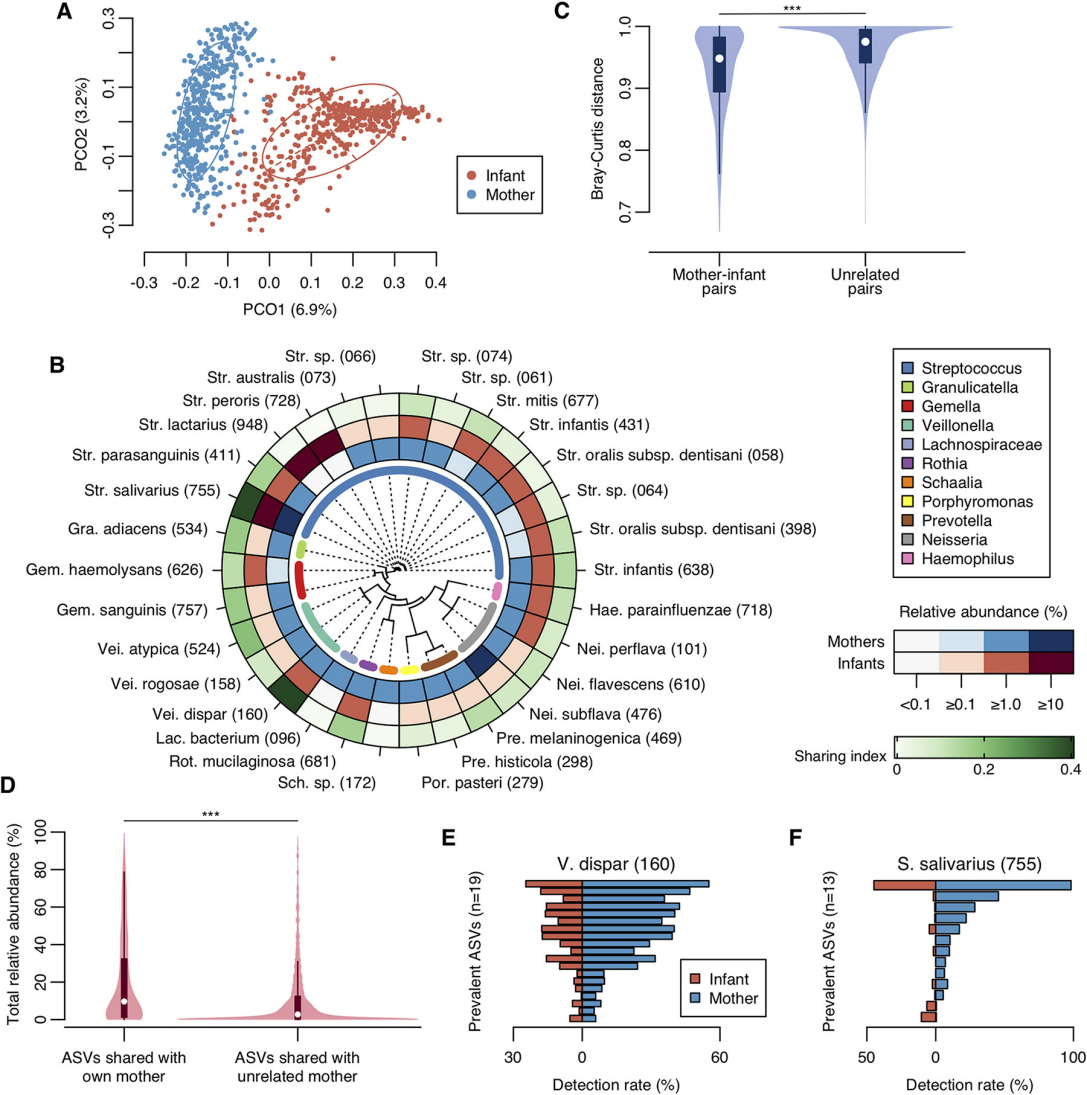
Microbial link of oral microbiota within mother-infant pairs. (A) PCoA plot of mother and infant samples based on the Bray-Curtis distance. Bacterial compositions of mother and infant samples are depicted using different colors. Intersections of broken lines indicate the center of gravity for each group. The ellipse covers 67% of the samples belonging to each group. (B) Mean relative abundance and sharing index of predominant OTUs. Thirty predominant OTUs with ≥1% relative abundance in either mothers or infants are ordered according to a phylogenetic tree. Mean relative abundance and sharing index of each predominant OTU are shown by color intensity. (C) Violin plot of the Bray-Curtis distances of mother-infant pairs (*n* = 448) and unrelated mother-infant pairs (*n* = 198,464). Significance was calculated using the Mann-Whitney U test. White dots indicate median values. ***, *P* < 0.001. (D) Violin plot of total relative abundance of ASVs shared with own mother (*n* = 448) and with unrelated mother (*n* = 198,464) in each infant. Significance was calculated using the Mann-Whitney U test. White dots indicate median values. ***, *P* < 0.001. (E, F) Distribution of prevalent ASVs corresponding to Veillonella dispar and Streptococcus salivarius. ASVs with ≥5% detection rate in either mothers or infants are shown.

First, we compared the overall bacterial compositions of mother and infant microbiota to identify the microbial link within mother-infant pairs. The maternal microbiota exhibited higher ASV diversity than the infant microbiota, and their bacterial compositions were distinctly different according to the principal coordinate analysis (PCoA) plot based on the Bray-Curtis distance (Fig. 1A and Fig. S1). The bacterial compositions of these microbiota were markedly different at the OTU level, as the maternal microbiota was dominated by Neisseria subflava HMT-476, Granulicatella adiacens HMT-534, and Prevotella melaninogenica HMT-469, whereas the infant microbiota was dominated by Streptococcus lactarius HMT-948 and Streptococcus peroris HMT-728 (Fig. 1B and Table S1 in the supplemental material). In contrast, Streptococcus salivarius HOT-755, Streptococcus infantis HMT-638, and Streptococcus parasanguinis HMT-411 prevailed in both groups. Moreover, the bacterial compositions in mother-infant pairs were significantly similar compared to the unrelated mother-infant pairs (Fig. 1C).

10.1128/mbio.03452-21.7 TABLE S1 Relative abundance of predominant OTUs Table S1, DOCX file, 0.1 MB. Copyright © 2022 Kageyama et al. 2022 Kageyama et al. https://creativecommons.org/licenses/by/4.0/ This content is distributed under the terms of the Creative Commons Attribution 4.0 International license.

To precisely evaluate microbial sharing between each mother and her infant, their bacterial compositions were compared at the ASV level, and shared ASVs that were detected in the mother and her own infant were identified in each mother-infant pair. Of 31.0 ± 14.1 observed ASVs in infants, a median of 4 ASVs (range of 0 to 40 ASVs) and a median of 16.7% over total number of ASVs observed in each infant (range 0.0 to 75.0%) were shared with the biological mother, and they accounted for a 9.7% median relative abundance in each infant microbiota (range 0.0 to 99.3%, Fig. 1D). These were significantly higher than the number (median of 2 ASVs), percentage (median 8.3%), and the total abundance (median 2.7%, Fig. 1D) of ASVs shared with unrelated mothers (all *P* < 0.001). Veillonella dispar HMT-160 and *S. salivarius* displayed a high sharing index (Fig. 1B). For instance, of 388 mothers and 268 infants with *V. dispar*, 189 mother-infant pairs had the same ASV corresponding to *V. dispar* (Table S2). Analysis of the distribution of prevalent ASVs with a detection rate of ≥5% in either mother or infant revealed that *V. dispar* showed similar distributions in mothers and infants (Fig. 1E). Although some ASVs of *S. salivarius* were prevalent only in mothers or infants, the leading ASV was common in both groups (Fig. 1F).

10.1128/mbio.03452-21.8 TABLE S2 Detections of predominant OTUs in mother and infant and their sharing indices Table S2, DOCX file, 0.1 MB. Copyright © 2022 Kageyama et al. 2022 Kageyama et al. https://creativecommons.org/licenses/by/4.0/ This content is distributed under the terms of the Creative Commons Attribution 4.0 International license.

### Clinical factors affecting oral microbial transmission from mother to infant.

**FIG 2:**
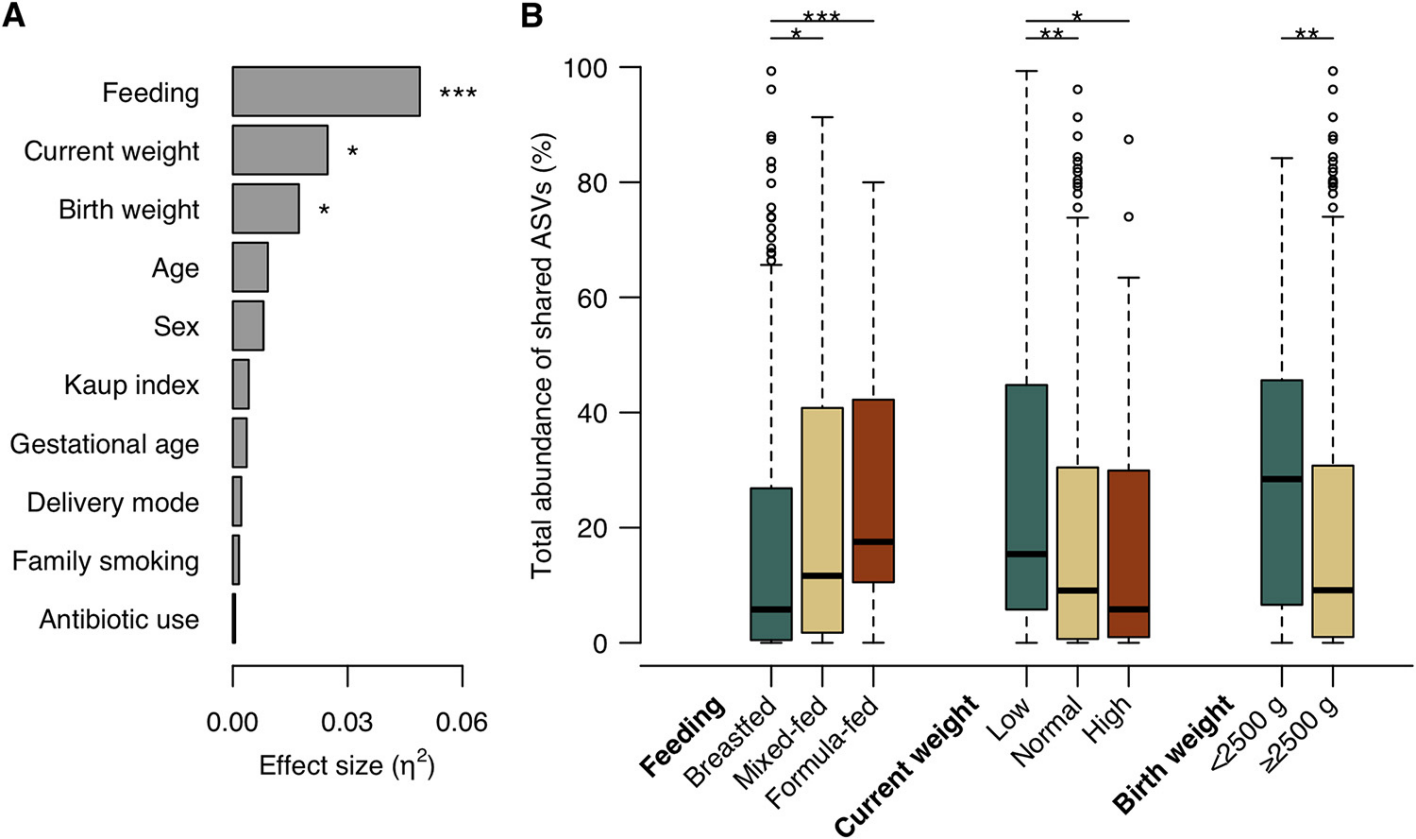
Influence factors on the oral microbial transmission from mother to her infant. (A) Effects of clinical factors on the total relative abundance of shared ASVs in infant microbiota. The effect size (η^2^) and significance of each factor were respectively calculated using the Kruskal-Wallis test. Obtained *P* values were adjusted using the FDR correction. *, *P* < 0.05; ***, *P* < 0.001. (B) Boxplot of the total abundance of shared ASVs in infant microbiota according to the factors with significance in the Kruskal-Wallis test. The significance was calculated using the Steel-Dwass test and the Mann-Whitney U test. *, *P* < 0.05; **, *P* < 0.01; ***, *P* < 0.001.

To explore the clinical factors affecting oral microbial transmission from mother to infant, we compared the total abundance of shared ASVs in infant microbiota according to clinical factors. The Kruskal-Wallis test demonstrated that feeding method, current weight, and birth weight were significantly associated with total relative abundance (Fig. 2A). On the one hand, in low-weight infants, the total abundance of shared ASVs was significantly higher than that in normal-weight infants (Fig. 2B). On the other hand, breastfed infants had a lower abundance of shared ASV than mixed- and formula-fed infants. There were no significant differences in abundance according to age, sex, Kaup index, gestational age, delivery mode, family smoking status, and antibiotic use (Fig. 2A and Fig. S2).

10.1128/mbio.03452-21.2 FIG S2 Boxplot of the total abundance of shared ASVs in infant microbiota according to the factors without significance in the Kruskal-Wallis test with the FDR correction. Download FIG S2, TIF file, 0.4 MB. Copyright © 2022 Kageyama et al. 2022 Kageyama et al. https://creativecommons.org/licenses/by/4.0/ This content is distributed under the terms of the Creative Commons Attribution 4.0 International license.

### Influence of maternal oral bacteria on infant oral microbiota.

**FIG 3:**
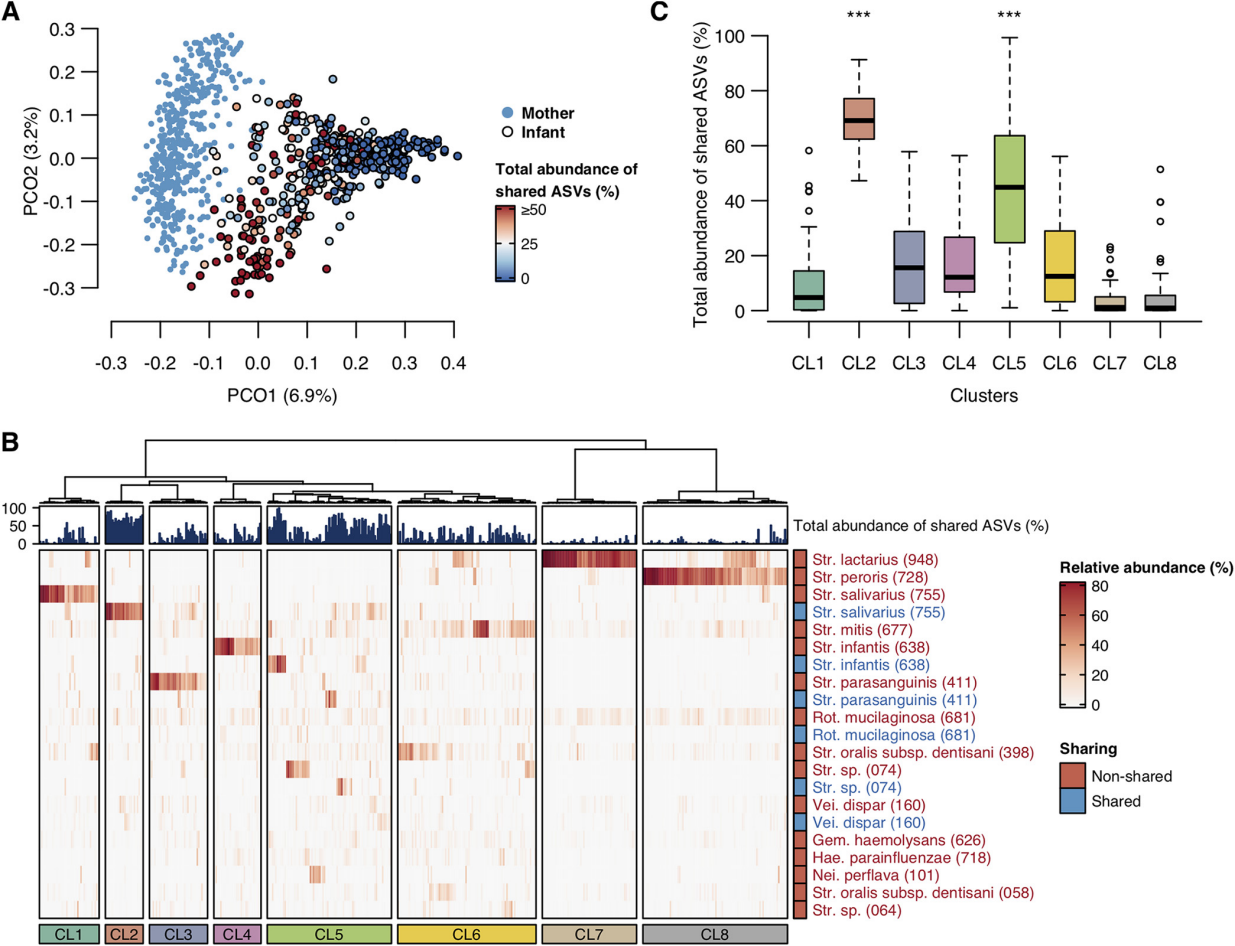
Relationship of maternal oral bacteria with infant oral microbiota. (A) PCoA plot based on the Bray-Curtis distance, with the total abundance of shared ASVs shown by gradient colors. Existence and nonexistence of dot outline indicate infant and mother samples, respectively. (B) Heatmap of bacterial composition of infant microbiota with bar plots showing total abundance of shared ASVs. The relative abundance of each OTU is represented by the color intensity. Cumulative abundances of shared ASVs with own mother are represented by blue color and cumulative abundances of nonshared ASVs with own mother are represented by red color, in each OTU. OTUs with a mean relative abundance of ≥1% are shown. The hierarchical clustering of infant microbiota is performed based on the Bray-Curtis distance. (C) Boxplot of the total relative abundance of shared ASVs in each cluster. Significance was calculated using the Steel-Dwass test. The asterisks indicate that they are significantly higher (*P* < 0.001) than all six other clusters.

Ultimately, we examined the influence of the total abundance of shared ASVs on infant oral microbiota. According to the PCoA plot, where gradient color expresses the total abundance of shared ASVs, infant microbiota with a higher abundance of shared ASVs were more similar to the mother microbiota (Fig. 3A). The hierarchical clustering approach, based on a modified OTU table that distinguished shared and nonshared ASVs, classified infant oral microbiota into eight clusters (Fig. 3B). The infants from CL2 and CL5 exhibited a significantly higher abundance of shared ASVs than the infants belonging to the other six clusters and demonstrated *S. salivarius-*dominant and mixed profiles (Fig. 3B and C). At the same time, the infants from CL7 and CL8 with low levels of shared ASV abundance demonstrated *S. lactarius-*dominant or *S. peroris-*dominant profiles.

## DISCUSSION

This study examined the oral microbiota of 448 mother-infant pairs and studied oral microbial transmission from mother to infant, which is considered a major acquisition route of infant microbiota. Full-length 16S rRNA gene amplicon analysis and the ASV approach identified that the infants shared 18.3% of total ASV number and 9.7% of relative abundance with their biological mother. In addition, this percentage and abundance were significantly higher than those of ASVs shared with unrelated mothers. These findings show that the infants acquired oral bacteria from their own mother’s oral microbiota, even at ASV levels, exhibiting as little as single-nucleotide differences. Moreover, the shared abundance varied widely, from 0.0% to 99.3% of the oral microbiota. These results suggest that the selective pressure against mother-derived bacteria, which would be constantly sourced by daily physical contact, varied widely among individuals.

The abundance of shared ASVs was strongly affected by the feeding method used. In particular, the breastfed infants had a lower abundance of shared ASVs compared to the formula-fed infants, and their bacterial composition was the most unrelated to the mother microbiota compared to the mixed- and formula-fed infants (Fig. S3). These results suggest that breastfeeding can be associated with a low maturity of infant oral microbiota due to the low acquisition of maternal bacteria. In fact, a similar tendency, where breastfeeding for a long duration was associated with a lower microbial maturity, was also observed in the gut microbiota (27). In addition to a nutritional role as an optimal food containing various nutrients, breast milk plays an immunological role as a gatekeeper while the infant immune system is immature by providing protective factors such as immunoglobulins, leukocytes, lactoferrin, and lysozyme (28, 29). The xanthine oxidase in breast milk has unique antibacterial activity within the neonatal mouth during breastfeeding and has also been reported to react with xanthine and hypoxanthine, which are abundant in neonatal saliva, and generate reactive oxygen species (30). It has also been suggested that the metabolic products of Streptococcus species which feed on human milk oligosaccharides, the third most abundant component of breast milk following lactose and lipid, might encourage the attachment and growth of selected oral commensal bacteria (31, 32). These conflicting functions of breast milk are likely to select for colonization by foreign bacteria, including mother-derived and infant-specific bacteria. Naturally, the differences in substrates provided by maternal milk and formula can also influence the infant oral microbiota profile. Further analyses focused on the regulatory mechanism must establish a novel strategy which controls the compositional balance and pathogenicity of oral microbiota.

10.1128/mbio.03452-21.3 FIG S3 PCoA plot based on the Bray-Curtis distance depicted by different colors according to feeding method. White dots in each violin plot indicate the median value. The distances between mother and infant with each feeding method were compared using the Steel-Dwass test. ***, *P* < 0.001. Download FIG S3, TIF file, 0.6 MB. Copyright © 2022 Kageyama et al. 2022 Kageyama et al. https://creativecommons.org/licenses/by/4.0/ This content is distributed under the terms of the Creative Commons Attribution 4.0 International license.

Higher acquisition of maternal oral bacteria was observed in physically immature infants with lower body weights and birth weights. This tendency remained consistent in a stratified analysis of feeding methods (Fig. S4). These results suggest that such high acquisition is not only attributed to the confounding effects of the slightly higher rate of formula-feeding in physically immature infants (Table S3). This result seems contradictory to the accepted notion that infant microbiota mature by acquiring mother-derived bacteria as the infant grows. Low birth weight is thought to impair immune system development and is reported as a risk factor for lower respiratory infection (33, 34), tuberculosis (35), and severe sepsis caused by respiratory infection or primary bacteremia (36). In fact, a recent study reported that low-birth-weight rats exhibited a lower inflammatory response to lipopolysaccharide instillation than normal-birth-weight rats (37). Although the influence of low weight on the oral mucosal immune system is unclear, this acquisition of maternal oral bacteria might be caused by dysregulated colonization due to prolonged immature immunity, rather than the construction of a normal microbial community.

10.1128/mbio.03452-21.4 FIG S4 Boxplot of the total abundance of shared ASVs in infant microbiota according to current weight (panel A) and birth weight (panel B). Subjects were stratified by feeding method. Significance was calculated using the Steel-Dwass test and the Mann-Whitney U test. *, *P* < 0.05. Download FIG S4, TIF file, 0.3 MB. Copyright © 2022 Kageyama et al. 2022 Kageyama et al. https://creativecommons.org/licenses/by/4.0/ This content is distributed under the terms of the Creative Commons Attribution 4.0 International license.

10.1128/mbio.03452-21.9 TABLE S3 Characteristics of infants in each feeding method Table S3, DOCX file, 0.1 MB. Copyright © 2022 Kageyama et al. 2022 Kageyama et al. https://creativecommons.org/licenses/by/4.0/ This content is distributed under the terms of the Creative Commons Attribution 4.0 International license.

We additionally examined the influence of the total abundance of shared ASVs on microbiota composition in infants. According to a hierarchical clustering approach based on a modified OTU table that distinguished shared and nonshared ASVs, infant oral microbiota was classified into several profiles. Most infants with very low abundance of shared ASVs demonstrated *S. lactarius-*dominant or *S. peroris-*dominant profiles. These species were rarely detected in the mother oral microbiota and disappeared by approximately 1.5 years of age in our previous study (13). Although few mothers had these species, only one sharing of *S. lactarius* was observed (Table S2). This finding emphasizes that *S. lactarius-*dominant and *S. peroris-*dominant profiles are specific to infancy and not derived from the maternal oral cavity. In contrast, infants with a higher abundance of shared ASVs showed the *S. salivarius*-dominant profile (CL2) and the mixed profile. *S. salivarius* is known as a major pioneer colonizer in infant microbiota (38). This result further suggests that maternal *S. salivarius* transmits to the infant oral cavity and becomes dominant in infant microbiota early in life. However, some infants had another type of *S. salivarius*-dominant profile which consisted of *S. salivarius* not derived from the maternal oral cavity (CL1). In fact, three differentially abundant ASVs in CL1, identified by the linear discriminant analysis effect size method (39) between eight clusters, corresponded to *S. salivarius* but were barely detectable from the mother microbiota (Fig. S5). Similar differences were also observed in the microbiota profiles dominated by *S. infantis*, *S. parasanguinis*, and Streptococcus species HMT 074. Thus, these results suggest that infant oral microbiota has several subtypes with different microbial origins, even if bacterial compositions seem to be the same at the OTU level. Although the origin of bacteria not derived from the maternal oral cavity remains unclear from our results, further examination of the longitudinal shift of similar-looking microbiota with different bacterial origins would provide us with novel and unique information about the impact of maternal or external bacteria on the development of the infant oral microbiota.

10.1128/mbio.03452-21.5 FIG S5 Boxplot of relative abundance of differentially abundant ASVs in CL1. Three ASVs were identified as differentially abundant ASVs for CL1 with ≥4.0 linear discriminant analysis scores by the linear discriminant analysis effect size method across eight clusters. All ASVs correspond to Streptococcus salivarius. Download FIG S5, TIF file, 0.2 MB. Copyright © 2022 Kageyama et al. 2022 Kageyama et al. https://creativecommons.org/licenses/by/4.0/ This content is distributed under the terms of the Creative Commons Attribution 4.0 International license.

Finally, we verified the influence of previously reported clinical factors (feeding method, delivery mode, family smoking, and antibiotic use) on the bacterial composition of infant microbiota. First, *P*. *melaninogenica* and *G. adiacens*, which were predominant in the microbiota of mothers, were significantly abundant in formula-fed infants (Fig. S6). In addition, their alpha diversities, based on the number of observed ASVs (43.9 ± 17.7), was significantly higher than they were in mixed-fed (32.1 ± 13.6) and breastfed (27.3 ± 11.3) infants. These results are consistent with the present study, finding that formula feeding is associated with high acquisition of maternal bacteria. In contrast, the effects of delivery mode, family smoking status, and antibiotic use were observed to be minimal in this study. Only *S. peroris* was more abundant in infants exposed to antibiotics within one month. Interestingly, although *S. peroris* is considered an early colonizer in infant oral mucosa along with *S. lactarius*, where the two are phylogenetically similar (40), they had opposite reactions to antibiotics. Further studies with a larger number of antibiotic-users are required to investigate the clinical relevance of differences in early colonizers due to antibiotic use.

10.1128/mbio.03452-21.6 FIG S6 Relative abundance of predominant OTUs according to feeding method, delivery mode, family smoking status, and antibiotics use. Predominant OTUs with ≥1% relative abundance in any group are shown for each clinical factor. These OTUs are listed in descending order of their abundance in the major group. Each bar plot shows the average of the relative abundance and the error bar denotes standard error. Significance was calculated using the Steel-Dwass test and the Mann-Whitney U test. *, *P* < 0.05, **, *P* < 0.01. Download FIG S6, TIF file, 0.7 MB. Copyright © 2022 Kageyama et al. 2022 Kageyama et al. https://creativecommons.org/licenses/by/4.0/ This content is distributed under the terms of the Creative Commons Attribution 4.0 International license.

This study had several limitations. First, we only investigated maternal oral microbiota as the major source of infant microbiota and disregarded other sources such as maternal skin and breastmilk, paternal oral cavity, and the environment. Several previous studies have reported the isolation of *S. lactarius* and *S. salivarius* from breast milk (40, 41). Indeed, all the infants in CL1 and 96.7% of the infants in CL7 were breastfed or mixed-fed in this study (Table S4). We should verify the detailed origins of infant oral bacteria not derived from the maternal oral cavity by investigating other maternal niches and environments. Second, our analyses relied on tongue microbiota, despite the fact that the maternal oral microbiota is likely to be transmitted into the infant oral cavity via saliva. However, previous studies have reported that the salivary microbiota is mainly composed of tongue microbes (42–44). Third, the clinical relevance of the acquisition of maternal oral bacteria could not be studied. A previous report has suggested that early exposure to parental oral microbes is associated with protection against early eczema development and asthma symptoms (45). The development of such diseases, considering the abundance of maternal oral bacteria in infant microbiota, should be further studied. Fourth, some information was lacking for the analysis, including the duration of breastfeeding until the checkup, the presence of teeth, pacifier use and cleaning practices, maternal oral health conditions (such as caries status and periodontal conditions), maternal antibiotic exposure, and total antibiotic exposure to the infants since birth. In particular, a previous study reported that salivary microbiota profiles in infants, which were determined by terminal-restriction fragment length polymorphism analysis, differed depending on whether the parents cleaned their pacifiers by sucking them (45). A thorough consideration of these limitations is necessary for further studies.

10.1128/mbio.03452-21.10 TABLE S4 Characteristics of infants with each bacterial profile Table S4, DOCX file, 0.03 MB. Copyright © 2022 Kageyama et al. 2022 Kageyama et al. https://creativecommons.org/licenses/by/4.0/ This content is distributed under the terms of the Creative Commons Attribution 4.0 International license.

In conclusion, the microbial transmission of maternal oral microbiota to infants was observed via full-length 16S rRNA gene amplicon analysis and the ASV approach, and the acquisition of maternal oral bacteria was markedly higher in formula-fed infants. In addition, distinguishing the origins of infant bacteria enabled the evaluation of bacterial compositions of infant oral microbiota with higher resolution. This novel information and approach can lay a foundation for further investigation of oral microbiota development, and could eventually lead to the establishment of novel preventive and treatment strategies to control bacterial balance and pathogenicity of oral microbiota. Further analyses should be performed to determine the associations between maternal bacterial abundance or origin-related bacterial profiles in infant oral microbiota and subsequent risk of oral and systemic diseases.

## MATERIALS AND METHODS

### Study subjects and data collection.

The subjects of this study were mother-infant pairs who visited health checkups for infants at 4 months of age in Higashi-ku, Fukuoka City, Japan, from January to March 2020. The checkup was composed of a medical interview, physical measurement, clinical examination by a doctor, and instruction on infant care. The city office sent invitation letters monthly to eligible infants aged around 4 months. Of 534 infants who underwent the checkup between January and March 2020, 448 mother-infant pairs, consisting of 448 infants and 444 mothers (including four mothers of twins), participated in the study. Written informed consent was obtained from all mothers. The ethics committee of Kyushu University approved the present study and the procedure for obtaining informed consent (approval no.: 2019-403). Samples were collected following a health checkup. Tongue swab samples were collected from all participating mothers and infants by scraping the tongue dorsum with a Puritan Hydraflock swab (Puritan Medical Products, Guilford, ME, USA). Collected samples were placed in sterile tubes with 200 μL of lysis buffer containing 9 mM Tris-HCl, 0.9 mM EDTA, and 0.1% sodium dodecyl sulfate. Samples were transported to our laboratory and stored at −80°C until further analysis. Clinical information such as sex, age (days), current weight and height, birth weight, gestational age, feeding method, delivery mode, antibiotic use within a month, and family smoking status were obtained from the clinical records of the checkup and our questionnaire.

### DNA extraction.

DNA was extracted using the bead-beating method. After the swab was discarded following centrifugation, 0.3 g zirconia-silica beads (bead size: 0.1 mm; BioSpec Products, Bartlesville, OK, USA) and one tungsten-carbide bead (bead size: 3 mm; Qiagen, Hilden, Germany) were added. The samples were heated at 90°C for 10 min and then agitated for 5 min using a cell disruptor (Disruptor Genie; Scientific Industries, Bohemia, NY, USA). After adding 150 μL of 1% sodium dodecyl sulfate and heating at 70°C for 10 min, the mixture was extracted with 500 μL of Tris-saturated phenol and again with 500 μL of phenol-chloroform-isoamyl alcohol (25:24:1). The nucleic acids were precipitated with 100% ethanol and sodium acetate and washed with 70% ethanol following centrifugation. The precipitated DNA was resuspended in 50 μL of TE buffer (10 mM Tris-HCl, 1 mM EDTA) and stored at −30°C until further analysis.

### Full-length 16S rRNA gene amplicon sequencing.

The full-length 16S rRNA gene containing all variable regions was amplified using the following primers with the sample-specific 8-base tag sequence: 8F (5′-AGA GTT TGA TYM TGG CTC AG-3′) and 1492R (5′-GGY TAC CTT GTT ACG ACT T-3′). PCR amplification was carried out using KOD DNA polymerase (Toyobo, Osaka, Japan) under the following cycling conditions: 98°C for 2 min, followed by 30 cycles of 98°C for 15 s, 60°C for 20 s, and 74°C for 90 s. Each PCR amplicon was purified using an Agencourt AMPure XP kit (Beckman Coulter, Brea, CA, USA), and equal amounts of purified amplicons were pooled. The pooled DNA was gel-purified using a Wizard SV Gel and PCR Clean-Up System (Promega, Madison, WI, USA). The purified amplicons were sequenced in four runs using the Sequel II Sequencing Kit 2.0 (Pacific Biosciences, Menlo Park, CA, USA) on a PacBio Sequel II (Pacific Biosciences), and the CCS reads with ≥3 full-pass subreads and ≥20 quality values were finally generated.

### Data analysis and taxonomy assignment.

The CCS reads were primary quality-checked using R software (version 3.6.2; R Foundation for Statistical Computing, Vienna, Austria) and were excluded from the analysis when they exhibited <1,000 bases or did not include the correct forward and reverse primer sequences. The remaining CCS reads were demultiplexed by examining the 8-base tag sequence at both ends, and the forward and reverse primer sequences were trimmed. The quality-checked CCS reads were further processed using the DADA2 pipeline (version 1.14.0), including quality-filtering, denoising (pseudo pooling), and chimera-filtering procedures with default settings for PacBio reads (except for the pooling option), and an ASV table was produced (25). Fourteen ASVs observed in the negative control were excluded from the ASV table and subsequent analysis as PCR contaminants. The taxonomy of each sequence variant was determined using BLAST (46) against 1,015 oral bacterial 16S rRNA gene sequences (16S rRNA RefSeq version 15.22) in eHOMD. Nearest-neighbor species with ≥98.5% identity were selected as candidates for each sequence. The taxonomy of sequences without hits was further determined using the RDP classifier with a minimum support threshold of 80% (47). We additionally constructed an OTU table from the ASV table by clustering ASVs assigned to the same reference sequence in eHOMD into an OTU.

### Statistical analysis.

All statistical analyses were performed using the R software. The dissimilarity of bacterial composition between mothers and infants was evaluated using the Bray-Curtis distance based on the square-root transformed ASV abundance data. The distances between the mother-infant pairs and unrelated mother-infant pairs were compared using the Mann-Whitney U test. A phylogenetic tree was constructed based on the reference sequences in eHOMD using the *ape* package in R (48). In this study, we defined ASVs that were detected in both the mother and her own infant as “shared ASVs” and calculated the number, percentage over total number, and total abundance of shared ASVs in each infant. Comparison of these values with those of ASVs shared with unrelated mothers was performed using the Mann-Whitney U test. To evaluate the transmissibility of predominant OTUs in mother-infant pairs, we calculated a sharing index for each OTU by dividing the number of mother-infant pairs with shared ASVs by the union of the number of mothers (including dual entry of mothers of twins) and the infants with the OTU. The sharing index ranges from 0.0 to 1.0, whereas a higher index expresses a higher rate of pairs with shared ASVs and pairs without the OTU on both. The Kaup index was calculated using the current weight and height (g/cm^2^ × 10). The current weight was categorized as low (below −1 standard deviation [SD] of the mean, <5,920.3 g), normal (within ±1 SD), or high (above +1 SD, >7,549.1 g). The total abundance of shared ASVs according to each clinical factor was compared using the Kruskal-Wallis test with calculating effect size (η^2^), and again using the Steel-Dwass test or the Mann-Whitney U test. *P* values obtained in the Kruskal-Wallis test were adjusted using Benjamini-Hochberg false discovery rate (FDR) correction for multiple testing. For a hierarchical clustering approach of infant microbiota, we constructed a modified OTU table by clustering shared ASVs and nonshared ASVs, respectively. Hierarchical clustering of infant microbiota was performed based on the Bray-Curtis distance. A heatmap based on the modified OTU table was plotted using the *ComplexHeatmap* package in R (49). The total abundance of shared ASVs in each cluster was compared using the Steel-Dwass test.

### Data availability.

The sequence data have been deposited in the DDBJ Sequence Read Archive under accession number DRA013064.
