# Supplementary figures and images for: High-Level Acquisition of Maternal Oral Bacteria in Formula-Fed Infant Oral Microbiota
Source: mBio. 2022 Jan 18;13(1):e03452-21. doi: 10.1128/mbio.03452-21 (PMC8764541; doi:10.1128/mbio.03452-21)

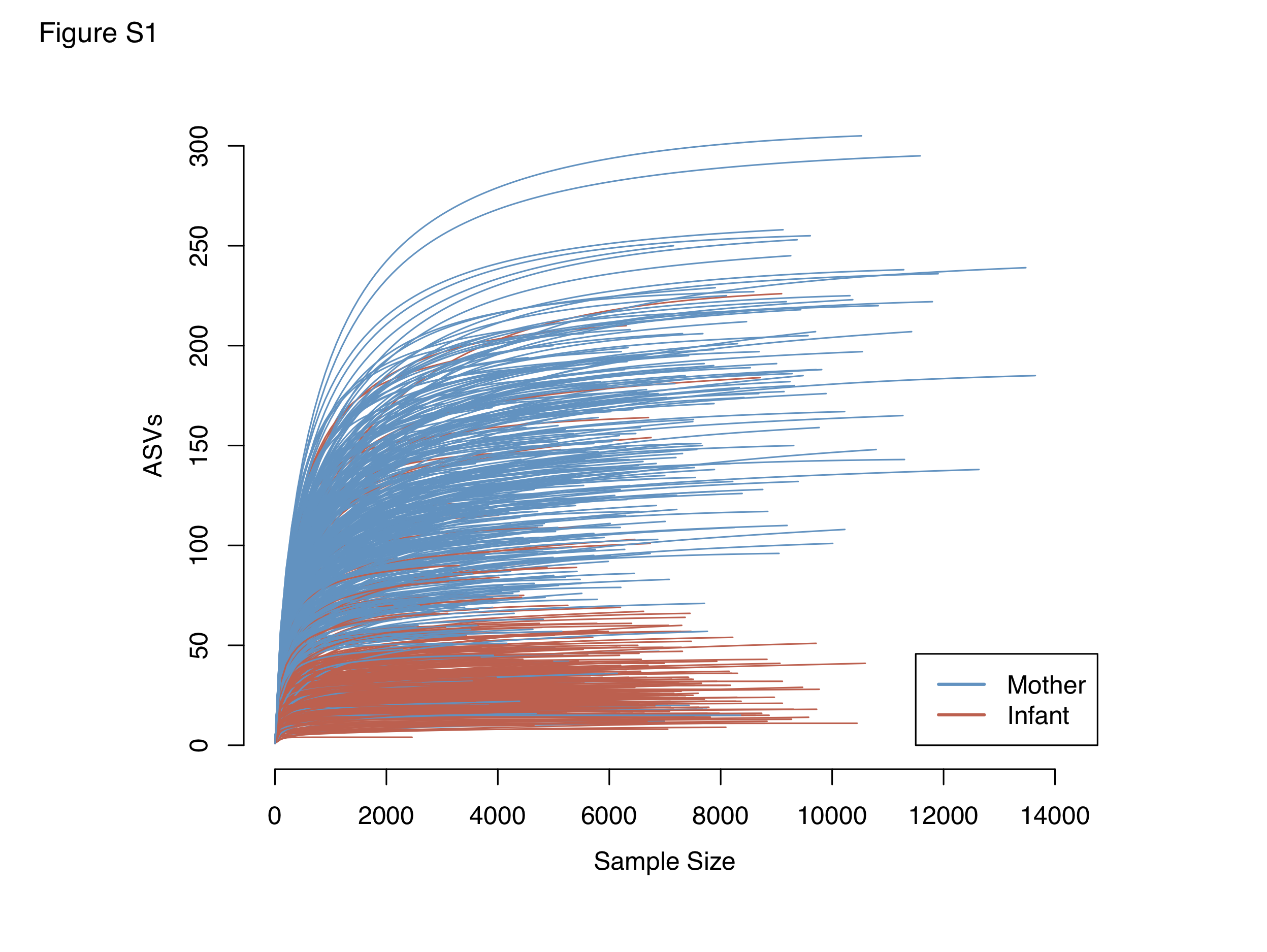

Supplement: FIG S1 [file mbio.03452-21-sf001.tif]

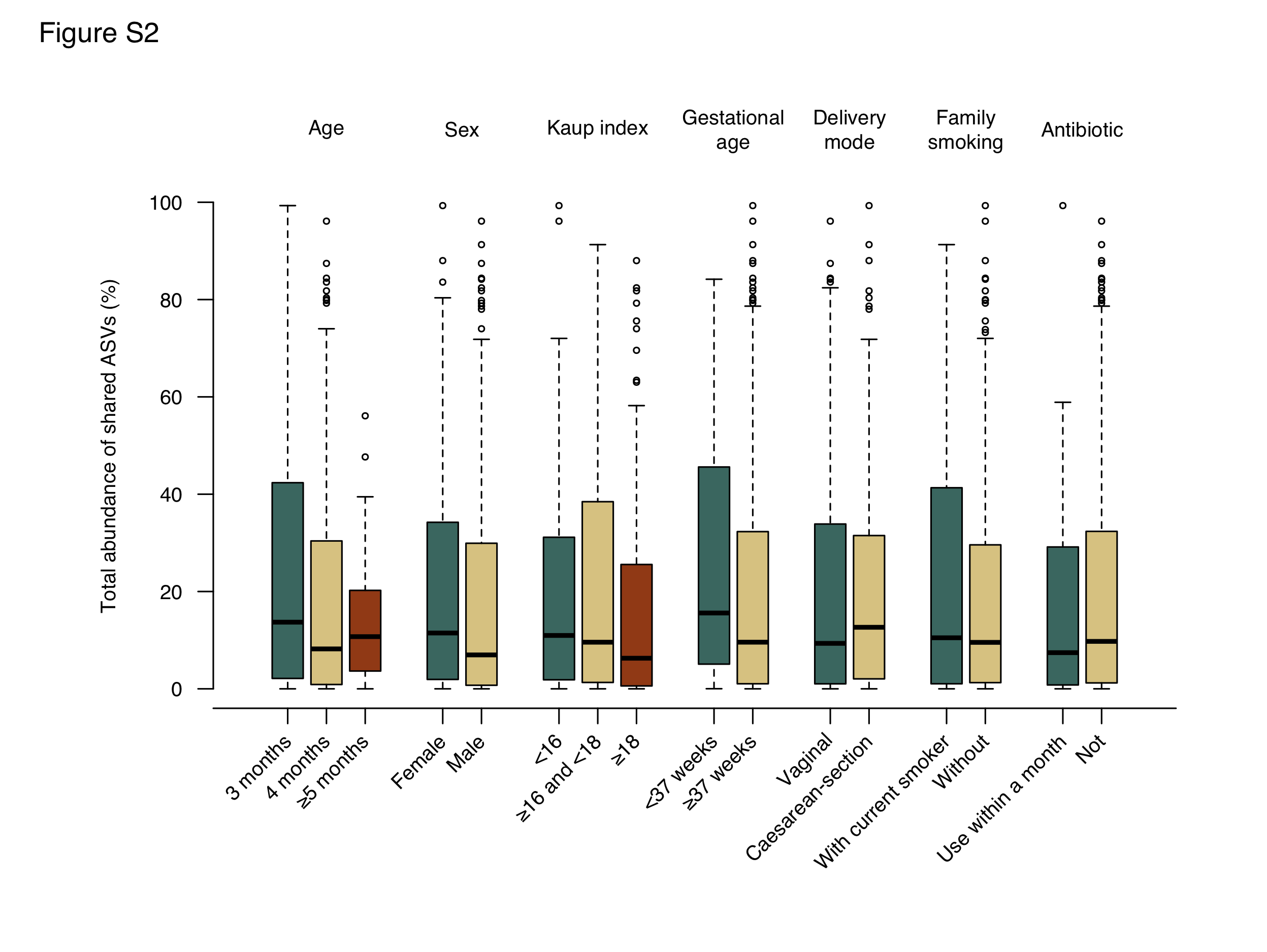

Supplement: FIG S2 [file mbio.03452-21-sf002.tif]

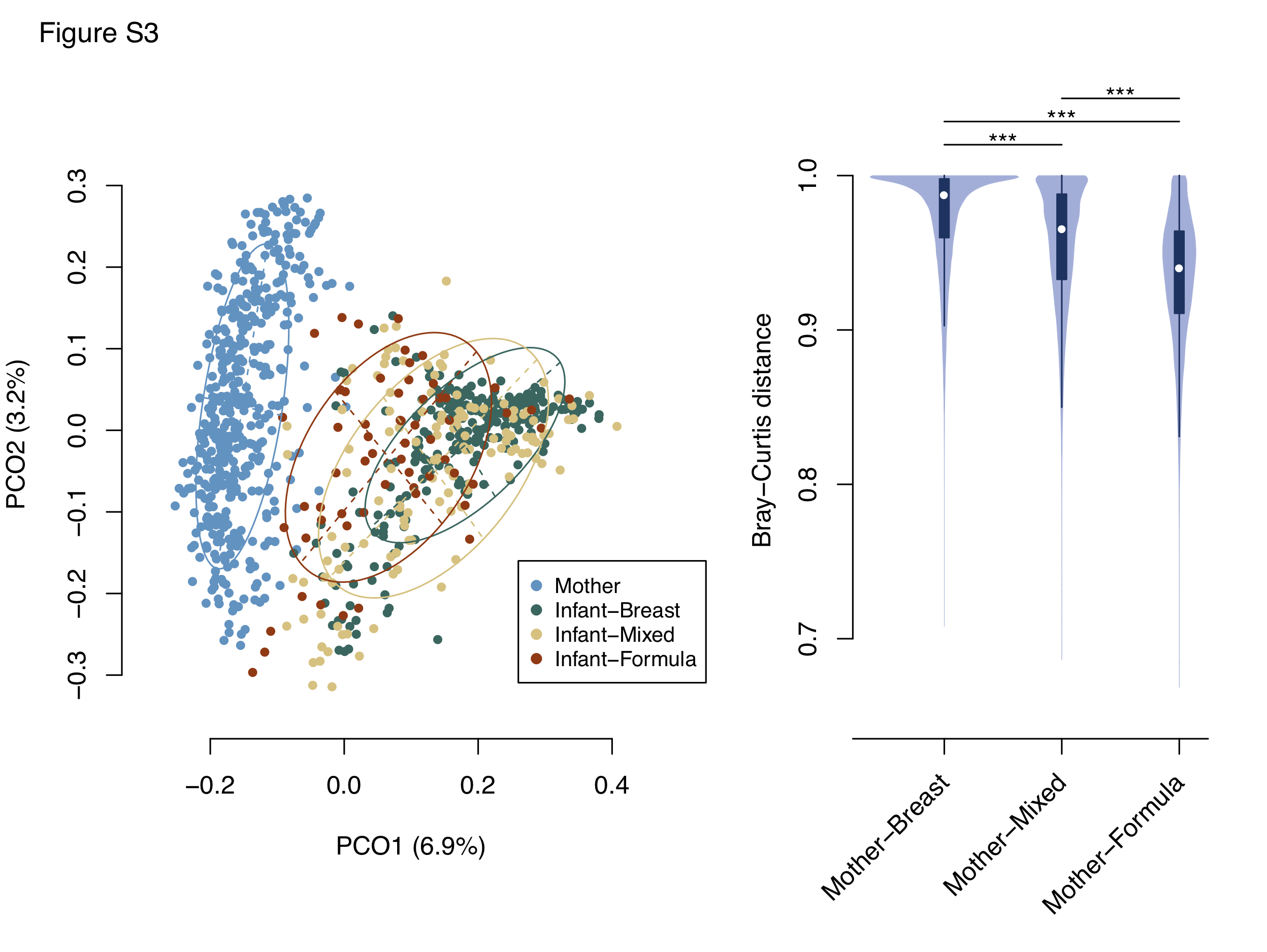

Supplement: FIG S3 [file mbio.03452-21-sf003.tif]

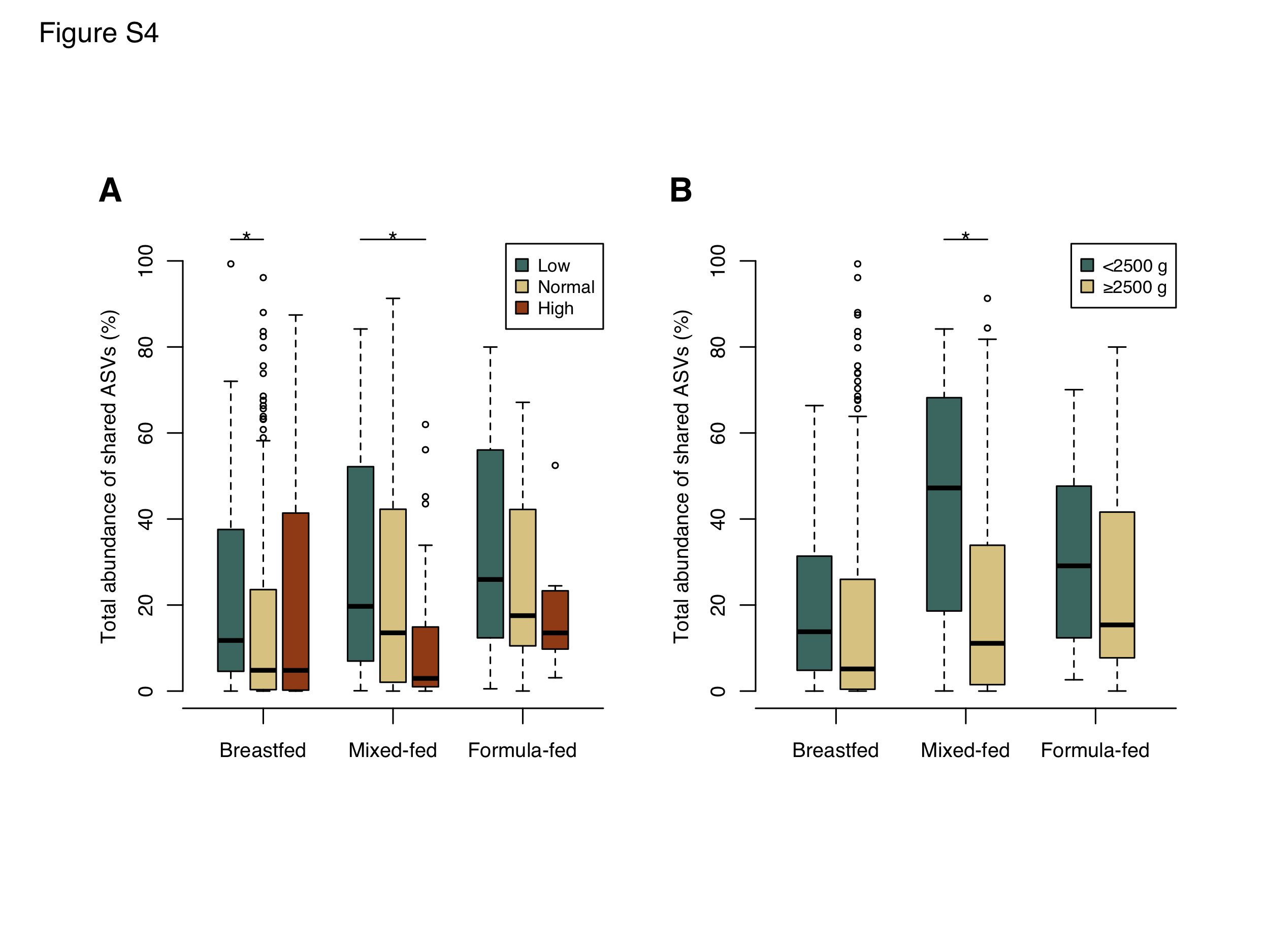

Supplement: FIG S4 [file mbio.03452-21-sf004.tif]

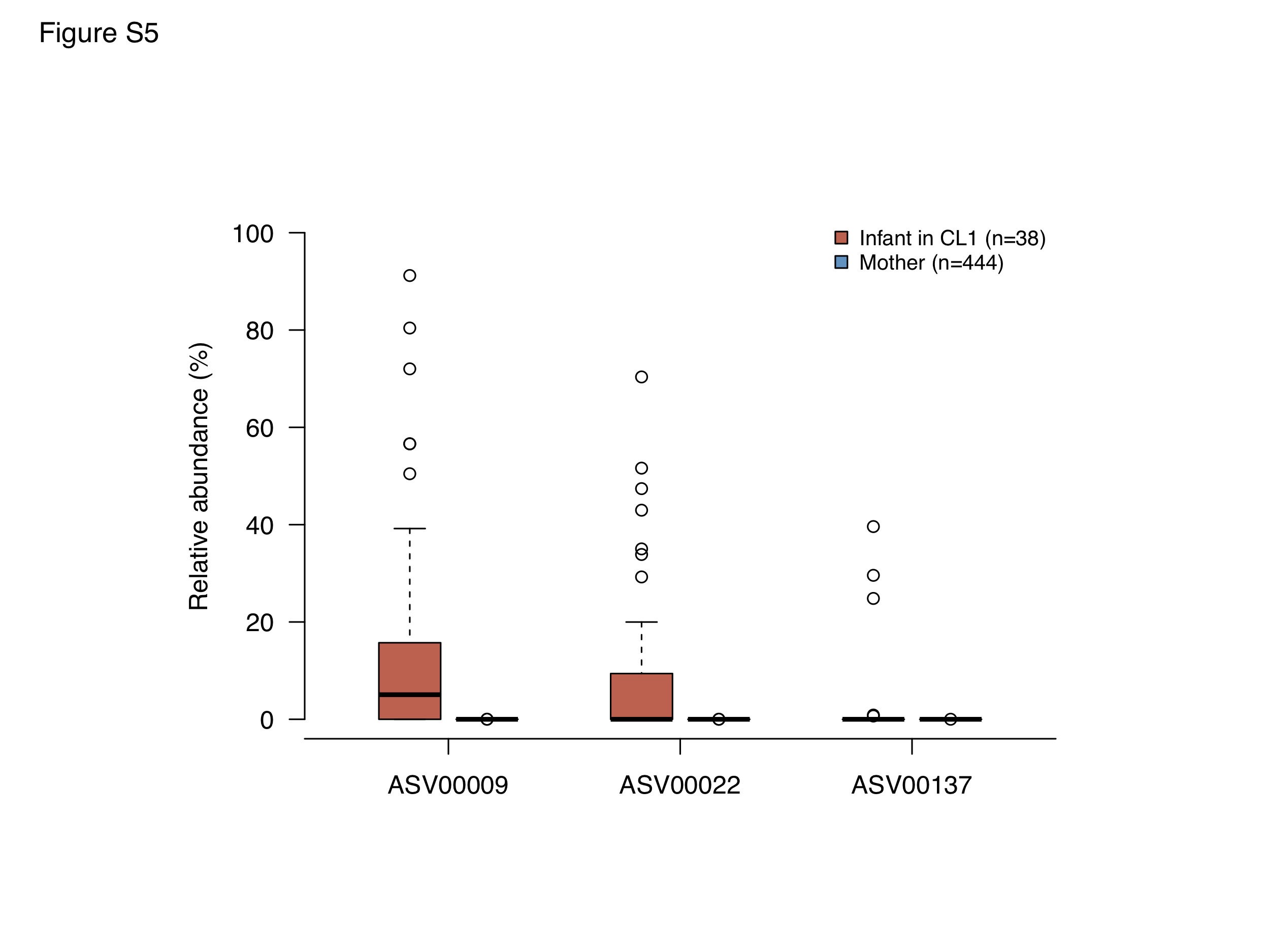

Supplement: FIG S5 [file mbio.03452-21-sf005.tif]

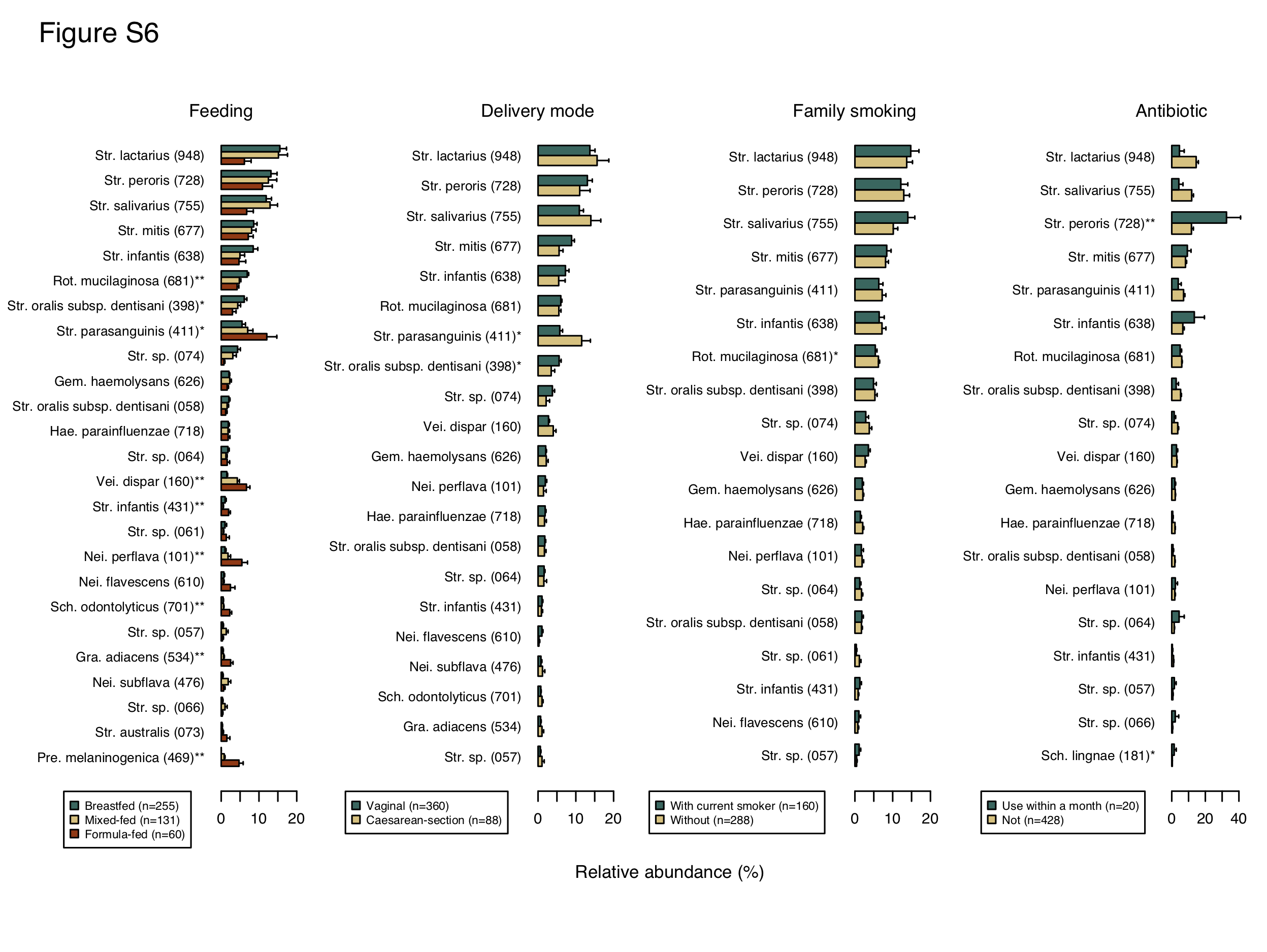

Supplement: FIG S6 [file mbio.03452-21-sf006.tif]
