# Supplementary material for: High-Level Acquisition of Maternal Oral Bacteria in Formula-Fed Infant Oral Microbiota
Source: mBio. 2022 Jan 18;13(1):e03452-21. doi: 10.1128/mbio.03452-21 (PMC8764541; doi:10.1128/mbio.03452-21)
Supplement: TABLE S1 [file mbio.03452-21-st001.docx]

**Table S1. Relative abundance of predominant OTUs.**

| Bacterial species corresponding to  predominant OTUs | Relative abundance (mean ± SD) | |  |
| --- | --- | --- | --- |
|  | Mother (n=444) | Infant (n=448) | P value |
| Dominant in mothers |  |  |  |
| *Neisseria subflava* (476) | 10.4 ± 15.5 | 0.8 ± 4.8 | <0.001 |
| *Granulicatella adiacens* (534) | 5.8 ± 4.0 | 0.8 ± 3.1 | <0.001 |
| *Prevotella melaninogenica* (469) | 3.0 ± 3.0 | 0.9 ± 3.8 | <0.001 |
| *Neisseria flavescens* (610) | 2.9 ± 8.4 | 0.9 ± 4.7 | <0.001 |
| *Streptococcus australis* (073) | 2.5 ± 3.8 | 0.4 ± 3.1 | <0.001 |
| *Gemella sanguinis* (757) | 2.0 ± 2.3 | 0.4 ± 0.9 | <0.001 |
| *Veillonella rogosae* (158) | 1.9 ± 2.3 | 0.2 ± 1.2 | <0.001 |
| *Streptococcus* sp. (061) | 1.7 ± 2.7 | 0.9 ± 5.8 | <0.001 |
| *Veillonella atypica* (524) | 1.6 ± 2.1 | 0.5 ± 1.2 | <0.001 |
| *Streptococcus* sp. (066) | 1.5 ± 2.5 | 0.5 ± 3.9 | <0.001 |
| *Porphyromonas pasteri* (279) | 1.4 ± 2.1 | 0.2 ± 0.7 | <0.001 |
| *Lachnospiraceae bacterium* (096) | 1.3 ± 3.1 | 0.1 ± 1.2 | <0.001 |
| *Schaalia* sp. (172) | 1.2 ± 1.7 | 0.0 ± 0.1 | <0.001 |
| *Prevotella histicola* (298) | 1.1 ± 2.0 | 0.2 ± 1.3 | <0.001 |
| Dominant in infants |  |  |  |
| *Streptococcus lactarius* (948) | 0.0 ± 0.0 | 14.1 ± 26.5 | <0.001 |
| *Streptococcus peroris* (728) | 0.0 ± 0.0 | 12.7 ± 24.8 | <0.001 |
| *Streptococcus mitis* (677) | 0.4 ± 0.7 | 8.2 ± 13.4 | <0.001 |
| *Streptococcus* *oralis* subsp. *dentisani* (398) | 0.2 ± 0.5 | 5.2 ± 9.8 | <0.001 |
| *Gemella* *haemolysans* (626) | 0.1 ± 0.2 | 2.1 ± 3.8 | <0.001 |
| *Streptococcus* sp. (064) | 0.1 ± 0.7 | 1.6 ± 5.0 | <0.001 |
| Dominant in common |  |  |  |
| *Streptococcus* *salivarius* (755) | 21.2 ± 16.8 | 11.5 ± 21.4 | <0.001 |
| *Streptococcus* *infantis* (638) | 5.1 ± 5.0 | 6.9 ± 17.4 | <0.001 |
| *Streptococcus* *parasanguinis* (411) | 4.2 ± 4.7 | 6.9 ± 15.9 | <0.001 |
| *Rothia* *mucilaginosa* (681) | 2.7 ± 2.9 | 5.9 ± 5.5 | <0.001 |
| *Veillonella* *dispar* (160) | 5.3 ± 5.1 | 3.0 ± 5.4 | <0.001 |
| *Neisseria* *perflava* (101) | 5.5 ± 11.0 | 1.8 ± 7.0 | <0.001 |
| *Streptococcus* sp. (074) | 1.1 ± 1.8 | 3.5 ± 10.5 | <0.001 |
| *Haemophilus* *parainfluenzae* (718) | 1.9 ± 2.6 | 1.8 ± 4.3 | <0.001 |
| *Streptococcus* *oralis* subsp. *dentisani* (058) | 1.2 ± 2.5 | 1.8 ± 4.4 | 0.002 |
| *Streptococcus* *infantis* (431) | 1.5 ± 2.3 | 1.0 ± 4.0 | <0.001 |

Thirty predominant OTUs with ≥1% of relative abundance in either mothers and infants are shown. Oral taxon IDs were given in parentheses following bacterial names. The significance was calculated using the Mann–Whitney U test and obtained P-values were adjusted using the FDR correction. SD, standard deviation.
