# Supplementary material for: High-Level Acquisition of Maternal Oral Bacteria in Formula-Fed Infant Oral Microbiota
Source: mBio. 2022 Jan 18;13(1):e03452-21. doi: 10.1128/mbio.03452-21 (PMC8764541; doi:10.1128/mbio.03452-21)
Supplement: TABLE S2 [file mbio.03452-21-st002.docx]

**Table S2. Detections of predominant OTUs in mother and infant and their sharing indices.**

|  | Mother (including dual entry of mothers of twins) | Infant | Pairs with shared ASVs | Sharing index |
| --- | --- | --- | --- | --- |
| *Veillonella* *dispar* (160) | 388 (391) | 268 | 189 | 0.402 |
| *Streptococcus* *salivarius* (755) | 441 (445) | 281 | 205 | 0.393 |
| *Veillonella* *atypica* (524) | 343 (345) | 187 | 91 | 0.206 |
| *Granulicatella* *adiacens* (534) | 443 (447) | 98 | 84 | 0.182 |
| *Gemella* *sanguinis* (757) | 431 (435) | 120 | 85 | 0.181 |
| *Rothia* *mucilaginosa* (681) | 420 (424) | 398 | 117 | 0.166 |
| *Streptococcus* *parasanguinis* (411) | 376 (380) | 222 | 83 | 0.16 |
| *Streptococcus* *infantis* (638) | 419 (423) | 173 | 73 | 0.14 |
| *Prevotella* *melaninogenica* (469) | 430 (434) | 108 | 64 | 0.134 |
| *Gemella* *haemolysans* (626) | 243 (245) | 368 | 70 | 0.129 |
| *Haemophilus* *parainfluenzae* (718) | 423 (427) | 199 | 67 | 0.12 |
| *Streptococcus* *oralis* subsp. *dentisani* (398) | 180 (182) | 314 | 53 | 0.12 |
| *Streptococcus* *mitis* (677) | 271 (273) | 404 | 70 | 0.115 |
| *Streptococcus* sp. (074) | 282 (284) | 100 | 37 | 0.107 |
| *Neisseria* *flavescens* (610) | 279 (282) | 62 | 33 | 0.106 |
| *Neisseria* *subflava* (476) | 302 (304) | 59 | 30 | 0.09 |
| *Veillonella* *rogosae* (158) | 369 (373) | 44 | 34 | 0.089 |
| *Neisseria* *perflava* (101) | 256 (258) | 87 | 27 | 0.085 |
| *Porphyromonas* *pasteri* (279) | 369 (373) | 68 | 26 | 0.063 |
| *Streptococcus* sp. (064) | 93 (94) | 159 | 12 | 0.05 |
| *Streptococcus* *infantis* (431) | 358 (360) | 90 | 20 | 0.047 |
| *Streptococcus* sp. (061) | 323 (326) | 40 | 16 | 0.046 |
| *Prevotella* *histicola* (298) | 293 (294) | 50 | 14 | 0.042 |
| *Streptococcus* *oralis* subsp. *dentisani* (058) | 248 (251) | 174 | 12 | 0.029 |
| *Streptococcus* *australis* (073) | 330 (333) | 31 | 8 | 0.022 |
| *Streptococcus* sp. (066) | 317 (319) | 34 | 6 | 0.017 |
| *Lachnospiraceae* *bacterium* (096) | 305 (305) | 6 | 2 | 0.006 |
| *Streptococcus* *lactarius* (948) | 5 (5) | 171 | 1 | 0.006 |
| *Schaalia* sp. (172) | 356 (358) | 15 | 2 | 0.005 |
| *Streptococcus* *peroris* (728) | 9 (9) | 156 | 0 | 0 |

﻿Thirty predominant OTUs with ≥1% of relative abundance in either mothers and infants are shown. Oral taxon IDs were given in parentheses following bacterial names.
