# Supplementary material for: High-Level Acquisition of Maternal Oral Bacteria in Formula-Fed Infant Oral Microbiota
Source: mBio. 2022 Jan 18;13(1):e03452-21. doi: 10.1128/mbio.03452-21 (PMC8764541; doi:10.1128/mbio.03452-21)
Supplement: TABLE S3 [file mbio.03452-21-st003.docx]

**Table S3. Characteristics of infants in each feeding method**

|  | Breastfeeding  (n=255) | Mixed-feeding  (n=131) | Formula-feeding  (n=60) | P value |
| --- | --- | --- | --- | --- |
| Sex |  |  |  | 0.097 |
| Female | 136 (53.3) | 58 (44.3) | 36 (60.0) |  |
| Male | 119 (46.7) | 73 (55.7) | 24 (40.0) |  |
| Age |  |  |  | 0.129 |
| 3 months | 68 (26.8) | 31 (24.0) | 14 (23.3) |  |
| 4 months | 177 (69.7) | 94 (72.9) | 39 (65.0) |  |
| ≥5 months | 9 (3.5) | 4 (3.1) | 7 (11.7) |  |
| Delivery mode |  |  |  | 0.097 |
| Vaginal | 214 (83.9) | 100 (76.3) | 45 (75.0) |  |
| Caesarean-section | 41 (16.1) | 31 (23.7) | 15 (25.0) |  |
| Antibiotic use |  |  |  | 0.235 |
| Not | 246 (96.5) | 125 (95.4) | 55 (91.7) |  |
| Use within a month | 9 (3.5) | 6 (4.6) | 5 (8.3) |  |
| Family smoking |  |  |  | 0.057 |
| Without | 176 (69.0) | 77 (58.8) | 34 (56.7) |  |
| With current smoker | 79 (31.0) | 54 (41.2) | 26 (43.3) |  |
| Gestational age |  |  |  | 0.098 |
| ≥37 weeks | 243 (96.0) | 125 (96.9) | 53 (89.8) |  |
| <37 weeks | 10 (4.0) | 4 (3.1) | 6 (10.2) |  |
| Birth weight |  |  |  | 0.050 |
| ≥2500 g | 238 (93.3) | 121 (92.4) | 50 (83.3) |  |
| <2500 g | 17 (6.7) | 10 (7.6) | 10 (16.7) |  |
| Current weight |  |  |  | 0.056 |
| Low | 28 (11.0) | 21 (16.3) | 13 (21.7) |  |
| Normal | 191 (75.2) | 82 (63.6) | 40 (66.7) |  |
| High | 35 (13.8) | 26 (20.2) | 7 (11.7) |  |
| Kaup index |  |  |  | 0.36 |
| <16 | 56 (22.0) | 28 (21.7) | 17 (28.3) |  |
| ≥16 and <18 | 144 (56.7) | 64 (49.6) | 32 (53.3) |  |
| ≥18 | 54 (21.3) | 37 (28.7) | 11 (18.3) |  |

﻿Subjects (%). The P-value was calculated using Fisher’s exact test.
