# Supplementary material for: High-Level Acquisition of Maternal Oral Bacteria in Formula-Fed Infant Oral Microbiota
Source: mBio. 2022 Jan 18;13(1):e03452-21. doi: 10.1128/mbio.03452-21 (PMC8764541; doi:10.1128/mbio.03452-21)
Supplement: TABLE S4 [file mbio.03452-21-st004.docx]

**Table S4. Characteristics of infants with each bacterial profile.**

|  | Cluster 1  (n=38) | Cluster 2  (n=24) | Cluster 3  (n=37) | Cluster 4  (n=30) | Cluster 5  (n=79) | Cluster 6  (n=88) | Cluster 7  (n=60) | Cluster 8  (n=92) | P value |
| --- | --- | --- | --- | --- | --- | --- | --- | --- | --- |
| Sex |  |  |  |  |  |  |  |  | 0.044 |
| Female | 17 (44.7) | 7 (29.2) | 15 (40.5) | 17 (56.7) | 41 (51.9) | 56 (63.6) | 27 (45.0) | 51 (55.4) |  |
| Male | 21 (55.3) | 17 (70.8) | 22 (59.5) | 13 (43.3) | 38 (48.1) | 32 (36.4) | 33 (55.0) | 41 (44.6) |  |
| Age |  |  |  |  |  |  |  |  | 0.052 |
| 3 months | 12 (31.6) | 10 (41.7) | 12 (32.4) | 4 (13.8) | 23 (29.5) | 20 (22.7) | 16 (27.1) | 16 (17.4) |  |
| 4 months | 26 (68.4) | 14 (58.3) | 24 (64.9) | 24 (82.8) | 54 (69.2) | 64 (72.7) | 41 (69.5) | 65 (70.7) |  |
| ≥5 months | 0 (0) | 0 (0) | 1 (2.7) | 1 (3.4) | 1 (1.3) | 4 (4.5) | 2 (3.4) | 11 (12) |  |
| Feeding method |  |  |  |  |  |  |  |  | 0.018 |
| Breastfeeding | 30 (78.9) | 11 (45.8) | 17 (47.2) | 18 (60.0) | 41 (51.9) | 48 (54.5) | 38 (63.3) | 52 (57.1) |  |
| Mixed-feeding | 8 (21.1) | 10 (41.7) | 10 (27.8) | 9 (30.0) | 22 (27.8) | 26 (29.5) | 20 (33.3) | 26 (28.6) |  |
| Formula-feeding | 0 (0) | 3 (12.5) | 9 (25) | 3 (10) | 16 (20.3) | 14 (15.9) | 2 (3.3) | 13 (14.3) |  |
| Delivery mode |  |  |  |  |  |  |  |  | 0.102 |
| Vaginal | 32 (84.2) | 19 (79.2) | 23 (62.2) | 23 (76.7) | 64 (81.0) | 76 (86.4) | 45 (75.0) | 78 (84.8) |  |
| Caesarean-section | 6 (15.8) | 5 (20.8) | 14 (37.8) | 7 (23.3) | 15 (19.0) | 12 (13.6) | 15 (25.0) | 14 (15.2) |  |
| Antibiotic use |  |  |  |  |  |  |  |  | 0.087 |
| Not | 38 (100) | 23 (95.8) | 37 (100) | 27 (90.0) | 76 (96.2) | 85 (96.6) | 59 (98.3) | 83 (90.2) |  |
| Use within a month | 0 (0) | 1 (4.2) | 0 (0) | 3 (10) | 3 (3.8) | 3 (3.4) | 1 (1.7) | 9 (9.8) |  |
| Family smoking |  |  |  |  |  |  |  |  | 0.352 |
| Without | 23 (60.5) | 10 (41.7) | 27 (73.0) | 20 (66.7) | 50 (63.3) | 61 (69.3) | 39 (65.0) | 58 (63.0) |  |
| With current smoker | 15 (39.5) | 14 (58.3) | 10 (27.0) | 10 (33.3) | 29 (36.7) | 27 (30.7) | 21 (35.0) | 34 (37.0) |  |
| Gestational age |  |  |  |  |  |  |  |  | 0.197 |
| ≥37 weeks | 36 (97.3) | 23 (95.8) | 31 (83.8) | 29 (96.7) | 76 (96.2) | 84 (97.7) | 57 (95.0) | 86 (95.6) |  |
| <37 weeks | 1 (2.7) | 1 (4.2) | 6 (16.2) | 1 (3.3) | 3 (3.8) | 2 (2.3) | 3 (5.0) | 4 (4.4) |  |
| Birth weight |  |  |  |  |  |  |  |  | 0.083 |
| ≥2500 g | 36 (94.7) | 20 (83.3) | 29 (78.4) | 28 (93.3) | 73 (92.4) | 82 (93.2) | 55 (91.7) | 88 (95.7) |  |
| <2500 g | 2 (5.3) | 4 (16.7) | 8 (21.6) | 2 (6.7) | 6 (7.6) | 6 (6.8) | 5 (8.3) | 4 (4.3) |  |
| Current weight |  |  |  |  |  |  |  |  | 0.073 |
| Low | 3 (7.9) | 6 (25.0) | 8 (21.6) | 7 (24.1) | 18 (23.1) | 8 (9.1) | 4 (6.8) | 8 (8.7) |  |
| Normal | 28 (73.7) | 14 (58.3) | 23 (62.2) | 18 (62.1) | 51 (65.4) | 68 (77.3) | 43 (72.9) | 69 (75.0) |  |
| High | 7 (18.4) | 4 (16.7) | 6 (16.2) | 4 (13.8) | 9 (11.5) | 12 (13.6) | 12 (20.3) | 15 (16.3) |  |
| Kaup index |  |  |  |  |  |  |  |  | 0.568 |
| <16 | 6 (15.8) | 6 (25.0) | 12 (32.4) | 8 (27.6) | 20 (25.6) | 24 (27.3) | 7 (11.9) | 18 (19.6) |  |
| ≥16 and <18 | 22 (57.9) | 13 (54.2) | 17 (45.9) | 14 (48.3) | 42 (53.8) | 47 (53.4) | 32 (54.2) | 54 (58.7) |  |
| ≥18 | 10 (26.3) | 5 (20.8) | 8 (21.6) | 7 (24.1) | 16 (20.5) | 17 (19.3) | 20 (33.9) | 20 (21.7) |  |

Subjects (%). The P-value was computed using Fisher’s exact test with Monte Carlo simulation.
